# Comparisons of Dementia Knowledge and Attitudes among the Youth and Older Adults: Insights from the Construal Level Theory Perspective

**DOI:** 10.3390/ijerph19041928

**Published:** 2022-02-09

**Authors:** Jianwei Wu, Sok-Man Leong, Sok-Leng Che, Iat-Kio Van, Yao-Chen Chuang

**Affiliations:** 1 Nursing and Health Education Research Centre, Kiang Wu Nursing College of Macau, Macao 999078, China; davidwu@kwnc.edu.mo (J.W.); shirley@kwnc.edu.mo (S.-L.C.); van@kwnc.edu.mo (I.-K.V.); 2 Research Management and Development Department, Kiang Wu Nursing College of Macau, Macao 999078, China; lsm@kwnc.edu.mo

**Keywords:** dementia, knowledge, attitudes, youth, older adults, Construal Level Theory

## Abstract

Based on Construal Level Theory (CLT), the youth and older adults have different psychological distances towards dementia that may lead to different dementia knowledge and attitudes. A cross-sectional survey among 239 youth and 62 older adults using a two-step sampling approach in Macao aimed to examine the hypothesis. Results showed older adults had a higher score of dementia knowledge (F_(1,299)_ = 45.692, *p* <0.001) but a lower score of dementia attitudes (F_(1,299)_ = 161.887, *p* <0.001) compared to the youth. Age group explained the majority of the variances in the hierarchical multiple regressions for dementia knowledge (R^2^ = 0.178, F = 9.059, *p* < 0.001) and for dementia attitudes (R^2^ = 0.399, F = 24.233, *p* < 0.001), which are β = 0.47 and −0.56, respectively. Thus, the hypothesis was supported and revealed an interesting pattern of dementia knowledge and attitudes among the youth and older adults. From the CLT perspective, the study implies that reducing and bridging the psychological distance of dementia would probably be an effective strategy to increase dementia awareness among young people, and intergenerational programs may be a good option to increase community acceptance and support for people with dementia.

## 1. Introduction

Dementia is a chronic neurodegenerative disease and a leading cause of disability and dependency among older people [1]. It can devastate the lives of affected individuals, their caregivers, and families. It is estimated that around 50 million people were living with dementia globally in 2018, and this number is expected to triple to 152 million by 2050 because of the aging population [2]. Therefore, dementia has become a globally prioritized health issue, and a global action plan was adopted by the World Health Organization (WHO) Member States in 2017 [3]. Macao is a Special Administrative Region of China, with a total population of 683,000, of which older people aged 65 years and above accounted for 12.9% in 2020 [4]. In addition, it is estimated that around six thousand people are currently living with dementia in Macao [5]. In response to the increasing dementia population, the government of Macao Special Administrative Region (SAR) launched a dementia policy in 2016, including a 10-year strategic framework to establish Dementia-Friendly Communities (DFCs) in Macao [6,7].

Increasing public knowledge of dementia is beneficial for detecting the first sign of dementia and seeking early diagnosis and treatment [8]. It also helps to tackle the stigma of dementia and to improve the quality of life and dignity of people with dementia, their caregivers, and families [3]. A systematic review revealed that the general public across the vast majority of studies had only fair-to-moderate knowledge and understanding, and the most common misconception was that dementia was a normal part of aging [9]. Therefore, raising awareness and friendliness towards dementia has been singled out as an important area of the global action plan regarding the public health response to dementia [3], and students are one of the proposed target groups in Macao’s dementia initiatives. Meanwhile, older people who are at risk of developing dementia are included as the stakeholders of DFCs. Previous studies revealed that dementia knowledge among community-dwelling adults, older adults, and high school students in Macao was insufficient [10,11]. Some research found differences in dementia knowledge and attitudes among different age groups, especially between the youth and older adults [12,13,14,15]. However, these differences have not yet been well explained from a theoretical perspective.

Construal Level Theory (CLT) is a theory in social psychology that describes the relationship between psychological distance and people’s perceptions towards objects or events [16,17]. Psychological distance is defined as “a subjective experience that something is closer or far away from the self, here, and now” [17]. The basic assumption of CLT is that people’s interpretation of an object or event relies on their psychological distance to the object or event, which means different psychological distances may lead to different perceptions and intentions towards one thing. Previous studies applied this theory to understand people’s perceptions and actions towards climate change, health-risk behavioral intentions, and knowledge and attitudes towards COVID-19 [18,19,20]. Since age is one of the dominant risk factors for dementia, the disease seems to be far away from younger people but closer to the older ones. Based on CLT, the youth and older adults have different psychological distances towards dementia; thus, we propose the hypothesis that the understanding and attitudes towards dementia between the youth and older adults are different. This study therefore aimed to examine the hypothesis and explain the results from the CLT perspective.

## 2. Materials and Methods

### 2.1. Research Design

A cross-sectional survey of the youth and older adults was conducted. A structured questionnaire (see Supplementary Materials) was used to assess the participants’ knowledge level and attitudes towards dementia in Macao.

### 2.2. Participants and Procedure

The target population in this study was young people aged from 15 to 30 and older adults aged 60 or above in Macao. There were a total of 49 high schools, 10 colleges, and nearly 50 community elderly centers throughout Macao. We recruited participants from these settings. A two-step sampling approach was used to ensure representativeness and minimize bias. Firstly, two high schools, three colleges, and two community elderly centers were purposely selected in different districts. Secondly, two classes were selected in each of the two high schools, and all the students in the selected classes were invited to participate in the present study. College students were surveyed by the selected institutions via email, whereas convenience sampling was used to recruit older adults in the selected community centers. Self-report method was used to collect data in the students’ group, while the interviewer-administered method was used with the older adults. We recruited 10 students from higher education institutions in Macao to train as investigators. All investigators were required to participate in a 10-hour training course regarding dementia knowledge and questionnaire practice. Data were collected from January to May of 2019.

### 2.3. Measurements

The questionnaire of this study included three sections: (1) socio-demographic data, (2) knowledge of dementia, and (3) attitudes towards dementia.

#### 2.3.1. Socio-Demographic Data

A socio-demographic questionnaire was developed to collect participants’ basic information, including gender, age, education, experience in caring for people with dementia, relatives or friends with dementia, and participation in dementia-related activities.

#### 2.3.2. Knowledge of Dementia

Dementia Knowledge Scale (DKS) contains 18 true/false items covering symptoms and risk factors domains to assess knowledge about dementia [13]. The Chinese version of DKS was translated following the forward-backward translation procedures [21]. Since the original scale has no published reliability and validity, the Chinese version scale was rated by 5 experts, including a neurologist, a geriatric nurse, a social worker, a physical therapist, and a specialist in public health, followed by a pilot study involving 48 participants. The Scale-level Content Validity Index (CVI) was 0.93, and the Cronbach’s α coefficient was between 0.74 and 0.84. A total score was calculated by summing the correct scores for each item, which ranged from 0 to 18; a higher total score indicating better knowledge.

#### 2.3.3. Attitudes towards Dementia

The Scale of Attitudes toward People with Dementia and their Care (APDC) was used to assess attitudes towards dementia [22]. The Chinese version of APDC contains 9 items with responses scored on a 5-point Likert scale ranging from 1 (strongly agree) to 5 (strongly disagree), covering interaction and care domains. The reliability of the Chinese version of APDC was established by Wu et al. [23], with a Cronbach’s α of 0.772. A total score ranged from 9 to 45, with higher scores indicating more positive attitudes.

### 2.4. Ethics

Ethical approval for this study was obtained from Kiang Wu Nursing College of Macau (Reference number: 2017OCT02). Permission to use the DKS and APDC was granted from the authors who developed them. Participants were provided with information sheets outlining the aim and process of this survey. Informed consent was obtained from participants in the present study, and participation was voluntary and confidential. All data collected were treated anonymously and confidentially.

### 2.5. Statistical Analysis

Data were checked for errors before double-entry computer input. Subsequently, they were analyzed through two stages with SPSS (Version 26) software [24]. In the first stage, descriptive statistics, such as mean, standard deviation, frequency, and percentage, were produced. The second stage involved inferential statistical analysis (one-way ANOVA and multiple linear regression analysis). Levene’s test was checked for homogeneity of variances before one-way ANOVA was conducted. Brown–Forsythe test was used when the variances across the different groups were not equal. Variance Inflation Factor (VIF) was used to check the presence of collinearity in multiple linear regression analysis, and it was suggested to cope with collinearity when VIF was greater than 10 [25]. Hierarchical multiple regression analysis was further used to identify the effects that the independent variables have on DKS and APDC scores. Statistical significance was based on *p*-value ≤ 0.05 in 2-tailed tests.

## 3. Results

### 3.1. Characteristics of Participants

A total of 301 valid questionnaires were returned for analysis, including 239 younger people aged between 15 and 30 and 62 older adults aged above 60. Table 1 summarizes the participants’ demographic characteristics of the youth group and the older adultsgroup. According to the results, more than half of the participants were female (59.1%), and the majority of education level was middle or high school (62.8%). The youth group aged from 15 to 30 (mean = 18.52, SD = 2.86), while the older adults group aged from 61 to 94 (mean = 78.55, SD = 8.95). Only a small proportion of participants reported that they had experience in caring for people with dementia (12.3%), had family members or relatives (17.3%) with dementia, or ever participated in dementia-related activities (17.3%). These two age groups showed significant differences in these characteristics.

### 3.2. More Sufficient Knowledge but Fewer Positive Attitudes towards Dementia in Older Adults Compared to the Youth

As shown in Table 2, the youth had a lower score of DKS both in symptoms and risk factors than older adults (F_(1,299)_ = 45.692, *p* <0.001). On the other hand, older adults had a lower score of APDC both in interaction and care than the youth (F_(1,299)_ = 161.887, *p* <0.001). The results showed that older adults had more knowledge, but the youth had more positive attitudes towards dementia. Thus, the hypothesis that the understanding and attitudes towards dementia between the youth and older adults are different was supported.

### 3.3. The Effect of Age Group on Dementia Knowledge and Attitudes

Hierarchical multiple regressions were conducted to examine the association of age groups with DKS and APDC. As shown in Table 3, education and participation in dementia-related activities and age group were significantly associated with DKS scores (F = 9.059, *p* < 0.001), explaining 17.8% of the total variance in dementia knowledge, and age group explained the majority of the variance in the model (β = 0.47). Moreover, gender, DKS scores, and age group were significantly associated with APDC scores (F = 24.233, *p* < 0.001), accounting for 39.9% of the total variance in dementia attitudes, and age group explained the majority of the variance in the model (β = −0.56) as well. Thus, the results suggested that the participants’ knowledge and attitude scores were significantly associated with different age groups.

**Figure 1:**
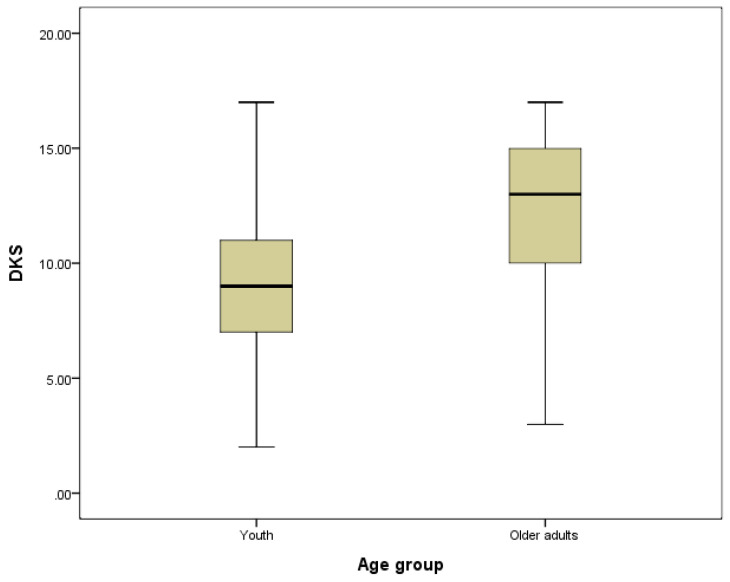
The difference in DKS between age groups.

**Figure 2:**
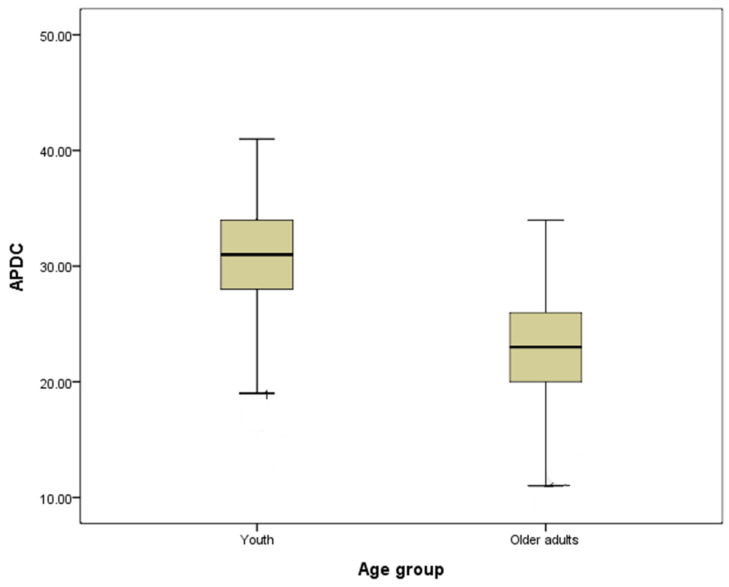
The difference in APDC between age groups.

Figure 1 and Figure 2 graphically illustrate the difference in DKS and APDC between the youth and older adults groups. These two box-plot figures also show that older adults had more dementia knowledge, but the youth had more positive attitudes towards dementia.

## 4. Discussion

### 4.1. Insights about the Differences in Dementia Knowledge and Attitudes among the Youth and Older Adults from the Perspective of Construal Level Theory

As shown in Table 2, the score of DKS was significantly higher in older adults than in high school and college students. Li et al. [12] found a similar result that the elderly group had more dementia knowledge than the youth group and the adult group in Shanghai communities. Moreover, a survey revealed that most high school students in Macao had insufficient knowledge [11]. The present study also revealed that older age was likely to be associated with more knowledge. Based on Construal Level Theory, an individual’s perceptions of an object or event relies on their psychological distance to the object or event. Since age is the biggest risk factor for dementia, older adults have a closer psychological distance towards dementia as compared to the youth. Previous studies have already demonstrated the role of psychological distance on an individual’s perception and motivation. For example, two experiments with a U.S. national opportunity sample found hazard proximity increased psychological proximity, weakly enhanced personal risk judgments concerning Zika transmission, and increased intentions of mosquito control [26]. Blauza et al. [18] found hypothetical distance (i.e., feeling to be likely affected by COVID-19) predicted participants’ affective, cognitive, and behavioral attitudes towards COVID-19. In another study, White et al. [27] showed psychological proximity increased the willingness for conforming to protective behaviors (i.e., paying for vaccines). Older people with closer psychological distance consider dementia with concrete levels of thinking and may worry more about developing dementia compared to the youth. Recent studies revealed that dementia worries are a widespread phenomenon in mid-life, and old age and is closely related to cognitive decline [28,29]. Nevertheless, moderate dementia worry can motivate people to access knowledge related to dementia and change lifestyle [30,31]. The data of participation in dementia-related activities (Table 1) show that older people actually participated in more dementia-related activities than college students and high school students (41.9% for older adults, 10.9% for young students). Similarly, another empirical research found that self-reported dementia worry was significantly associated with higher levels of dementia knowledge [32]. Apart from age groups, Table 3 also showed that education and participation in dementia-related activities were positively associated with dementia knowledge. It is reasonable that people can reduce the psychological distance by education and participation in dementia-related activities, thus increasing dementia knowledge. It was consistent with previous research in Construal Level Theory. Previous studies revealed that education and experiences can increase the psychological distance between individuals and climate change and between organizations and their members. For example, those who reported flood experience were more willing to engage in energy conservation to mitigate climate change [33].

On the other hand, older adults’ attitudes towards dementia were found to be less positive than the student groups in the present study. Marcinkiewicz and Reid [13] found a similar result: that a larger proportion of older people thought people with dementia can often be violent and aggressive than other age groups and found younger people express a more optimistic view about caring for someone with dementia as well. From the perspective of Construal Level Theory, older people who are at risk of developing dementia have closer psychological distance and consider dementia with concrete levels of thinking, which may lead to greater fear towards dementia compared to the youth. Ebert, Kulibert, and McFadden [34] found that greater personhood-based knowledge instead of biomedical knowledge and less personal dementia fear significantly predicted higher levels of social comfort with people living with dementia. Researchers surmised that people who feel a greater threat of developing dementia may project their own anxiety and distress onto the people living with dementia, which leads to discomfort or avoidance [28,35]. Some research actually found that older people would feel more fearful and more uncomfortable with friends or relatives with dementia [34,36,37]. Thus, the differences in dementia knowledge and attitudes among the youth and older adults in the present study can be well explained using Construal Level Theory and provide insights about raising public awareness of dementia.

### 4.2. Implications

The findings of the present study have several theoretical and practical implications. To begin with, this study revealed an interesting pattern of dementia knowledge and attitudes among the youth and older adults from the Construal Level Theory perspective. Although prior research indicated the differences in dementia knowledge and attitudes among different age groups [12,13,14,15], these differences have not yet been well explained from a theoretical perspective. Construal Level Theory is a new theoretical perspective to understand dementia awareness and attitudes among the different-aged populations. As a leading contemporary theory of mental construal in consumer science, CLT is most commonly used to explain consumer behavior [38]. Thus, this study also broadens the application of CLT.

Additionally, the findings of an interesting pattern of dementia knowledge and attitudes among the youth and older adults may provide educators and practitioners with insights about raising public awareness and establishing Dementia-Friendly Communities. Firstly, the study implies that reducing and bridging the psychological distance of dementia would probably be an effective strategy to increase dementia awareness among young people, such as the international campaign of “Let’s Talk About Dementia” in 2019, the 2020 World Alzheimer’s Month launched by ADI, and creating a dementia-friendly generation as advocated by the Alzheimer’s Society. Although there is a lack of empirical research on the application of CTL in dementia awareness, researchers have produced evidence that using this strategy to reduce the psychological distance of climate change is effective [39]. Secondly, the study shows more sufficient knowledge but fewer positive attitudes towards dementia in older adults compared to the youth. It implies that attitudes towards dementia and specific dementia knowledge may not have a positive linear relationship. Another study also found that greater personhood-based knowledge instead of biomedical knowledge significantly predicted higher levels of social comfort with people living with dementia [34]. Hence, educators and practitioners ought to take a balance between biomedical knowledge and attitudes in the promotion of dementia-friendly community initiatives. In addition, the study implies that reducing and bridging the psychological distance of dementia would probably be an effective strategy to increase dementia awareness. As discussed above, it is reasonable that people can reduce the psychological distance by education and participation in dementia-related activities, especially among younger people, who have more psychological distance from the disease. Therefore, some intergenerational programs, such as frequent creative participation activities in which memory care residents interact with adolescents, may be a good option to increase community acceptance and support for people with dementia [40].

### 4.3. Limitations and Future Research

Our study had some limitations. Firstly, a cross-sectional design of the present study cannot reveal causal relationships but only associations between variables. Secondly, we were not able to conduct random sampling because of limited conditions. Even with a two-step sampling approach, it may still slightly affect the representativeness of samples. Lastly, the current research assumed participants’ psychological distance towards dementia based on their risk of getting dementia and assumed participants’ feelings towards dementia based on other literature rather than measuring participants’ psychological distance and feelings directly. Even though Construal Level Theory and previous studies have demonstrated strong support, the direct measure of psychological distance and feelings would have strengthened our understanding of the underlying mechanism of the findings. Thus, a further investigation needs to be conducted in future research. As other study found that greater personhood-based knowledge instead of biomedical knowledge significantly predicted higher levels of social comfort with people living with dementia, we suggest to add the measure of personhood-based knowledge in the future study. Furthermore, some new methods, such as Propensity Score Matching, are proposed for aiming to reduce confounding bias and improve causal inference in observational studies [41].

## 5. Conclusions

This study compared dementia knowledge and attitudes among the youth and older adults from a new perspective. From the CLT perspective, older people with closer psychological distance consider dementia with concrete levels of thinking and may worry more about developing dementia compared to the youth, while moderate dementia worry can motivate people to access dementia knowledge but may lead to discomfort or avoidance of people with dementia. The results showed an interesting pattern that there is more sufficient knowledge but fewer positive attitudes towards dementia in older adults compared to the youth. Identifying this interesting pattern could be helpful for practitioners and educators in anticipating promoting needs, which can provide implications for the further development of DFCs. Moreover, CLT is indeed a new theoretical perspective to understand dementia awareness among the different aged populations, which broadens the application of CLT.

## Figures and Tables

**Table 1:** Demographic characteristics and comparison of the youth and older adults groups (*n* = 301).

Characteristics	Summary Statistics	Age Groups		F Value or χ^2^ Value	*p*-Value
		Youth (*n* = 239)	Older Adults (*n* = 62)		
Age (years; mean ± SD, range)	30.89 ± 24.78, 15–94	18.52 ± 2.86, 15–30	78.55 ± 8.95, 61–94	2716.190 ^a^	<0.001
Gender, *n* (%)				17.275 ^b^	<0.001
Male	123 (40.9)	112 (46.9)	11 (17.7)		
Female	178 (59.1)	127 (53.1)	51 (82.3)		
Education, *n* (%)				231.258 ^b^	<0.001
Primary or below	50 (16.6)	0 (0)	50 (80.6)		
Middle or high school	189 (62.8)	179 (74.9)	10 (16.1)		
Associate degree or above	62 (20.6)	60 (25.1)	2 (3.2)		
Experience in caring for people with dementia, *n* (%)				5.451 ^b^	0.020
Yes	37 (12.3)	24 (10.0)	13 (21.0)		
No	264 (87.7)	215 (90.0)	49 (79.0)		
Family and relatives with dementia, *n* (%)					
Yes	52 (17.3)	39 (16.3)	13 (21.0)	0.745 ^b^	0.388
No	249 (82.7)	200 (83.7)	49 (79.0)		
Friends with dementia, *n* (%)				36.509 ^b^	<0.001
Yes	23 (7.6)	7(2.9)	16 (25.8)		
No	278 (92.4)	231 (97.1)	46 (74.2)		
Participation in dementia-related activities				33.225 ^b^	<0.001
Yes	52 (17.3)	26 (10.9)	26 (41.9)		
No	249 (82.7)	213 (89.1)	36 (58.1)		

^a^ Statistics were based on Brown–Forsythe test, ^b^ Statistics were based on Pearson’s chi-square test.

**Table 2:** Comparisons of DKS and APDC between the youth and older adults by one-way ANOVA (*n* = 301).

Scales/Domains	Mean ± SD	Age Groups		* F * Value
		Youth (*n* = 239) Mean ± SD	Old Adults (*n* = 62) Mean ± SD	
DKS (18 items)	9.89 ± 3.59	9.22 ± 3.39	12.45 ± 3.19	45.692 ***
Symptoms (8 items)	5.56 ± 1.85	5.40 ± 1.83	6.18 ± 1.84	8.836 **
Risk factors (10 items)	4.33 ± 2.44	3.82 ± 2.28	6.27 ± 2.00	59.673 ***
APDC (9 items)	29.48 ± 5.90	31.26 ± 4.65	22.63 ± 5.16	161.887 ***
Interaction (5 items)	18.39 ± 4.03	19.54 ± 3.13	13.94 ± 4.03	103.559 ^#,^***
Care (4 items)	11.10 ± 2.59	11.72 ± 2.28	8.69 ± 2.29	86.439 ***

DKS, Dementia Knowledge Scale; APDC, the Scale of Attitudes toward People with Dementia and their Care. ^#^ Statistics were based on Brown–Forsythe test, ** *p* < 0.01, *** *p* < 0.001.

**Table 3:** Multiple linear regression analysis for DKS and APDC (*n* = 301).

Variables			DKS		APDC					
	Model 1		Model 2		Model 1		Model 2		Model 3	
	B (95%CI)	t Value (β)	B (95%CI)	t Value (β)	B (95%CI)	t Value (β)	B (95%CI)	t Value (β)	B (95% CI)	t Value (β)
(Constant)	10.58 (9.01~12.16)	13.24 ***	6.84 (4.88~8.8)	6.86 ***	20.43 (18.05~22.82)	16.86 ***	24.83 (21.92~27.73)	16.83 ***	30.07 (27.1~33.04)	19.913 ***
Gender (Female = 1)	0.51 (−0.32~1.35)	1.21 (0.07)	0.01 (−0.8~0.82)	0.02 (0.00)	0.29 (−0.98~1.55)	0.45 (0.02)	0.5 (−0.72~1.72)	0.81 (0.04)	1.39 (0.25~2.53)	2.41 * (0.12)
Education	−0.7 (−1.38~−0.02)	=−2.03 * (−0.12)	0.99 (0.12~1.85)	2.25 * (0.17)	4.42 (3.39~5.46)	8.44 *** (0.46)	4.13 (3.13~5.13)	8.13 *** (0.43)	0.94 (−0.28~2.17)	1.52 (0.10)
Experience in caring for people with dementia (Yes = 1)	0.01 (−1.32~1.33)	0.01 (0.00)	−0.41 (−1.67~0.86)	−0.63 (−0.04)	−0.22 (−2.23~1.79)	−0.21 (−0.01)	−0.22 (−2.15~1.72)	−0.22 (−0.01)	0.59 (−1.19~2.38)	0.66 (0.03)
Family and relatives with dementia (Yes = 1)	0.61 (−0.53~1.74)	1.06 (0.06)	0.52 (−0.56~1.6)	0.95 (0.06)	−0.02 (−1.74~1.7)	−0.02 (−0.00)	0.23 (−1.43~1.9)	0.28 (0.02)	0.29 (−1.23~1.81)	0.38 (0.02)
Friends with dementia (Yes = 1)	−0.34 (−1.91~1.23)	−0.43 (−0.03)	−1.34 (−2.87~0.19)	−1.72 (−0.10)	−0.54 (−2.93~1.84)	−0.45 (−0.02)	−0.68 (−2.98~1.61)	−0.59 (−0.03)	1.36 (−0.8~3.52)	1.24 (0.06)
Participation in dementia-related activities (Yes = 1)	2.02 (0.9~3.15)	3.54 *** (0.21)	1.36 (0.27~2.45)	2.45 * (0.14)	−0.43 (−2.13~1.27)	−0.50 (−0.03)	0.41 (−1.27~2.09)	0.48 (0.03)	1.31 (−0.24~2.86)	1.67 (0.08)
DKS			-----	-----			−0.42 (−0.58~−0.25)	−4.87 *** (−0.25)	−0.21 (−0.37~−0.05)	0.3367
Age group (old adults = 1)			4.14 (2.73~5.55)	5.78 *** (0.47)					−8.18 (−10.27~−6.08)	−7.69 *** (−0.56)
R^2^	0.084		0.178		0.219		0.277		0.399	
F	4.498 ***		9.059 ***		13.718 ***		16.061 ***		24.233 ***	
ΔR^2^	0.084		0.094		0.219		0.059		0.122	
ΔF	4.498 ***		33.448 ***		13.718 ***		23.752 ***		59.132 ***	

B, unstandardized coefficients; CI, confidence interval; β, standardized coefficients; * *p* < 0.05, *** *p* < 0.001.

## Data Availability

Data available on request due to ethical considerations. The data presented in this study are available on request from the corresponding author.

## Supplementary Materials

The following supporting information can be downloaded at: https://www.mdpi.com/article/10.3390/ijerph19041928/s1, Questions from the questionnaire (Reference number: 2017OCT02).

## Abbreviations

ADI	Alzheimer’s Disease International
ANOVA	Analysis of variance
APDC	The Scale of Attitudes toward People with Dementia and their Care
CLT	Construal Level Theory
CVI	Content Validity Index
DFCs	Dementia-Friendly Communities
DKS	Dementia Knowledge Scale
Macao SAR	Macao Special Administrative Region
VIF	Variance Inflation Factor
WHO	World Health Organization
