# Supplementary material for: Comparisons of Dementia Knowledge and Attitudes among the Youth and Older Adults: Insights from the Construal Level Theory Perspective
Source: Int J Environ Res Public Health. 2022 Feb 9;19(4):1928. doi: 10.3390/ijerph19041928 (PMC8872372; doi:10.3390/ijerph19041928)
Supplement: Supplementary file 1 [file ijerph-19-01928-s001.zip › ijerph-1567661-supplementary.pdf]

Questions from the questionnaire (Reference number: 2017OCT02)

1.Knowledge of Dementia

1.1Knowledge of Symptoms of Dementia

	Items	True	False	Don't know
1.	Difficulty in recognising people			
2.	Putting things in the wrong place (eg. putting shoes in the refrigerator)			
3.	Slower thinking			
4.	Losing track of time (eg. confused about day and night)			
5.	Feeling lost in new places			
6.	Feeling extremely tired			
7.	Losing their temper easily			
8.	Feeling depressed			

1.2 Knowledge of Risk Factors of Dementia

	Items	True	False	Don't know
1.	There is nothing anyone can do to reduce their risks of getting dementia			
2.	High blood pressure increases your chances of getting dementia			
3.	If one of your parents gets dementia, you are more likely to get it too			
4.	Smoking has nothing to do with dementia			
5.	People who drink heavily are more likely to get dementia			
6.	People who take regular physical exercise are less likely to get dementia			
7.	People who experience depression are more likely to get dementia			
8.	People with diabetes are more likely to get dementia			
9.	Obese people are more likely to get dementia			
10.	People who are socially active are less likely to get dementia			

2. Attitudes towards Dementia

	Items	Strongly agree	Agree	Neither agree nor disagree	Disagree	Strongly disagree
1.	There comes a time when all you can do for someone with dementia is keep them clean, healthy and safe					
2.	Once they have dementia, the person you knew eventually disappears					
3.	As soon as someone is diagnosed with dementia, they are not treated like a thinking human being any more					
4.	For people with really bad dementia life is not worth living					
5.	People with dementia are like children and need to be cared for as you would a child					
6.	It is better for people with dementia and their families if they are cared for in a residential or nursing home					
7.	There is little or no benefit to be gained from telling someone they have dementia					
8.	People who have just been diagnosed with dementia are unable to make decisions about their own care					
9.	There is no point in trying to talk to people with dementia as they won't be able to understand					

3. Personal Information

1. Gender：☐Male ☐Female
2. Are you Macao resident：☐Yes ☐No
3. Age：__________years-old
4. Education level：
   ☐No formal education ☐Primary school or below ☐Junior high school ☐High school
   ☐Associate
   ☐Bachelor
   ☐Master's or above
5. Your current occupation：☐Police officer ☐Bank staff ☐Bus driver ☐Taxi driver ☐ Tourist bus driver☐ Supermarket Staff ☐ Superintendent ☐ High school student ☐ College student ☐ Retiree ☐Other______________
   6a. Work schedules：☐Full-time ☐Part-time
6. Experience in caring for people with dementia：☐Yes ☐No
7. Family and relatives with dementia：☐Yes ☐No
8. Friends with dementia：☐Yes ☐No
9. Participation in dementia-related activities：☐Yes ☐No
   If yes：
   10a. What type (multiple choice) ：☐Talk ☐Workshop ☐exhibition ☐Visit ☐Other_____
   10b. Organiser (multiple choice) ：☐ Social service organisation ☐ Professional organisation ☐ Government ☐Other____________
   10c. When was the last time you attended
   ☐Within 1 year ☐1 to less than 3 year ☐3 to less than 5 years ☐ 5 years or over

》》》》》》》》》》》》》》》》》》》》》》》》》》》》》》 The end 《《《《《《《《《《《《《《《《《《《《《《《《《《《《《《
